# Quantitative evaluation of adhesion severity around subscapularis and its relationship with shoulder range of motion in frozen shoulder and rotator cuff disorder: an observational study using dynamic ultrasonography

**DOI:** 10.1016/j.jseint.2024.01.016

**Published:** 2024-02-21

**Authors:** Mizuki Fujiwara, Norma Hermawan, Takuya Suenaga, Yoshihiro Hagiwara, Yoshifumi Saijo

**Affiliations:** a Tohoku University Graduate School of Biomedical Engineering, Sendai, Japan; b Department of Rehabilitation, Sendai Hospital of East Japan Railway Company, Sendai, Japan; c Institut Teknologi Sepuluh Nopember, Surabaya, Indonesia; d Department of Orthopedic Surgery, Tohoku University School of Medicine, Sendai, Japan

**Keywords:** Frozen shoulder, Rotator cuff disorder, Adhesion, Ultrasonography coracohumeral ligament, Subscapularis, Range of motion

## Abstract

**Background:**

This study aimed to evaluate the severity of adhesion between muscles in the shoulder joint using dynamic ultrasonography and to confirm whether adhesions cause range of motion (ROM) restrictions.

**Methods:**

Twenty-four shoulders from 15 frozen shoulder patients and 24 shoulders from 18 rotator cuff disorder patients were enrolled. We obtained ultrasound video sequences of the subscapularis (SSC) and deltoid muscles during shoulder external rotation. The mean stretching velocities of the deltoid and SSC were subsequently analyzed using a personal computer. If adhesions occurred between both muscles, the deltoid was stretched more vigorously, and we calculated mean stretching velocity of the deltoid / SSC as adhesion severity. The coracohumeral ligament thickness was measured using the same images. Shoulder ROM was measured by using a universal goniometer.

**Results:**

The intraclass correlation coefficients (1.1) and (2.1) of the adhesion severity measurements were 0.85 and 0.91, respectively. Multiple linear regression analysis revealed that the adhesion severity is a significant predictor for external rotation ROM in the rotator cuff disorder group (R^2^ = 0.44, F = 10.1, *P* < .01, t = −2.9), while coracohumeral ligament thickness predicts ROM in the frozen shoulder group (R^2^ = 0.28, F = 5.5, *P* = .01, t = −3.0).

**Conclusion:**

The proposed method is reliable. Muscle adhesion causes ROM restriction of the shoulder joint. The primary cause of shoulder ROM restriction differed between the diagnostic groups.

Frozen shoulder (FS), also referred to as adhesive capsulitis, is a common musculoskeletal condition that affects 2%-5% of the general population.^11^ The main symptoms of FS include night pain, painful shoulder motion, and active and passive shoulder range of motion (ROM) restrictions. ROM restriction interferes with various activities of daily living, such as perineal care and hair-combining.^18^

Joint capsule thickening is the primary cause of shoulder ROM restriction.^19^ The most characteristic manifestation of FS is a thickened coracohumeral ligament (CHL) that restricts external rotation ROM.^11^ However, in our cases, some patients experience ROM restrictions even after joint capsule and CHL resection under arthroscopy,^9^^,^^10^ suggesting that other factors contribute to ROM restriction.

A possible cause is the adhesion between the soft tissues observed around symptomatic knee and wrist joints.^1^^,^^21^ Contrary to its diagnostic name, "Adhesive capsulitis," Ha'eri and Maitland^8^ observed no intra-articular (ie, pericapsular) adhesions in FS and suggested the existence of an extra-articular cause. A dynamic ultrasound study revealed that subscapularis (SSC) tendon gliding was limited during external rotation movement in the FS.^22^ The SSC is a primary active stabilizer for external rotation^17^ and is adjacent to the deltoid, which is the largest muscle in the shoulder joint. Some surgeons have proposed arthroscopic resection of adhesions around the SSC tendon to restore the ROM.^20^ Therefore, we believe that extra-articular (ie, SSC and deltoid) adhesions are present and restrict ROM in FS.

To evaluate the adhesion severity, we implemented the MATLAB (MathWorks, Natick, MA, USA) code based on previous research,^12^ which calculates the velocity of the stretched tissues using speckle-tracking ultrasonography. The speckle-tracking method is an ultrasonography-specific tracking method that is widely used in the cardiovascular domain to evaluate myocardial function.^3^ This method enables the analysis of spatial displacement within a region of interest (ROI) via block matching between images.

Although the deltoid is not strongly stretched by external rotation (Supplementary Video S1), if adhesions occur between muscles, it would be stretched more vigorously (Supplementary Video S1). This can generate an additional elastic force, resulting in ROM restrictions. Therefore, we analyzed the stretched velocity of the deltoid (V_Delt_) relative to the SSC (V_SSC_) during shoulder external rotation and represented the results as the “Deltoid–Subscapularis Adhesion Index” (DSAI: DSAI = V_Delt_/V_SSC_). Higher DSAI values indicate that the deltoid is more vigorously stretched by adhering to the SSC. Since it was predicted that image sequence evaluation would be more challenging than static image evaluation, the first step in this study was to measure reliability.

The primary objective of this study was to confirm the reliability of the new method for evaluating adhesion severity in the shoulder joint. In addition, we investigated whether adhesions other than CHL thickening are a cause of shoulder ROM restriction. We evaluated them in not only FS but also rotator cuff disorder (RCD). RCD-related ROM restriction is generally distinguished from idiopathic FS. Although the pain and disuse can cause ROM restriction in RCD, there is no clear evidence for the etiological difference of shoulder restriction between FS and RCD. Thus, we compared the ultrasound findings between the diagnostic conditions to confirm whether the etiological factors differed.

## Materials and methods

This cross-sectional observational study was conducted between January 2020 and August 2021.

The institutional review board approved this study (IRB number 2019-1-778), and all participants provided written informed consent.

### Participants

Successive patients who needed shoulder operation (arthroscopic capsular release or rotator cuff repair) were assigned to the FS or RCD groups. Diagnostic criteria for FS included shoulder external rotation ROM of 30° or less, shoulder flexion ROM of 130° or less, idiopathic onset, no supraspinatus tear detected on magnetic resonance imaging,^5^ and no radiological abnormality. RCD was defined as a full-thickness supraspinatus tear detected on magnetic resonance imaging.^5^ The exclusion criteria included full-thickness SSC tear detected by ultrasound^2^ or magnetic resonance imaging,^6^ inability to perform active shoulder external rotation up to neutral rotation, 1 month or less after onset, traumatic onset, and history of shoulder infection, fracture, and surgery. We excluded shoulders with external rotation ROM <0° because we could not obtain dynamic ultrasonography with sufficient quality to analyze adhesion severity. The image sequences from these samples generated many image artifacts and significant variations in DSAI between frames.

### Outcome measures

Ultrasonography and shoulder ROM measurement were performed on the same day.

For adhesion severity measurements, we used a commercial ultrasound system (Aplio i800 TUS-AI800; Canon Medical Systems, Tokyo, Japan) and an 18-MHz linear array probe (PLI-1805BX; Toshiba, Tokyo, Japan). Two examiners trained in musculoskeletal ultrasonography performed dynamic ultrasonography. The probe was placed on the anterior side of the glenohumeral joint to obtain a clear view of the coracoid process and longitudinal view of the SSC.

**Figure 1:**
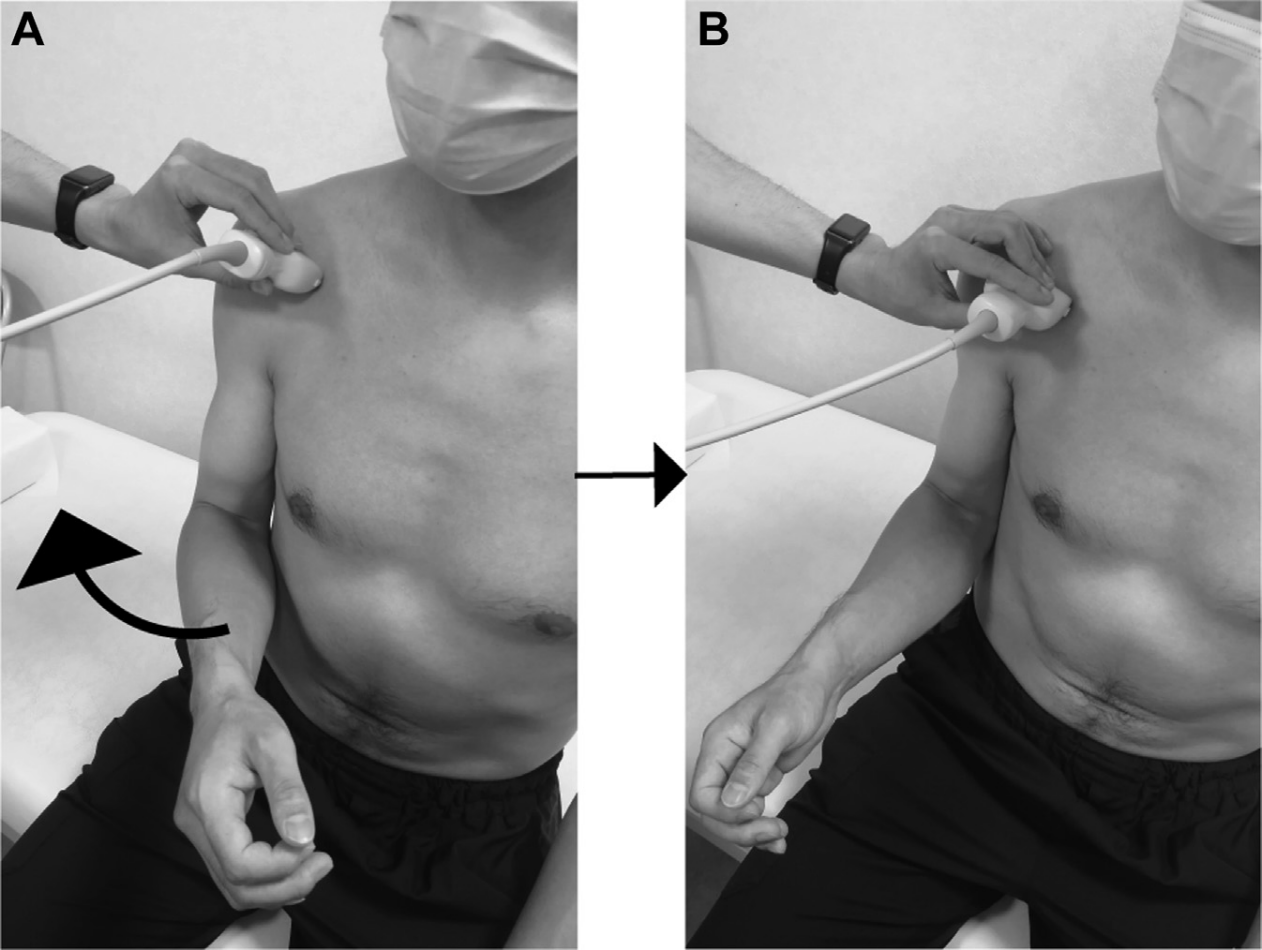
Dynamic ultrasonography setting. (**A**): Initial position of the motion task, (**B**): Terminal position of the motion task.

Each participant performed shoulder external rotation from 45° internal rotation with the arm at the side to a neutral rotation position in approximately 3 seconds with 90° elbow flexion in the sitting position (Fig. 1). We used a digital metronome application for the smartphone (Metronome Beats; Stone Kick Limited, London, UK) to maintain constant movement speed. We recorded image sequences twice in each subject at 32 fps.

After recording the dynamic ultrasonography, we analyzed the DSAI using the following procedure.

1. The video was played frame-by-frame to determine the initial and terminal frames of the motion. The boundary between the deltoid and SSC was also confirmed.
2. In the initial frame, a rectangular ROI was placed where the diagonal line overlapped the boundary line of the deltoid and SSC.
3. V_Delt_ and V_SSC_ were analyzed as velocities in the direction parallel to the diagonal of the ROI, with the distal side being positive (Fig. 2).

**Figure 2:**
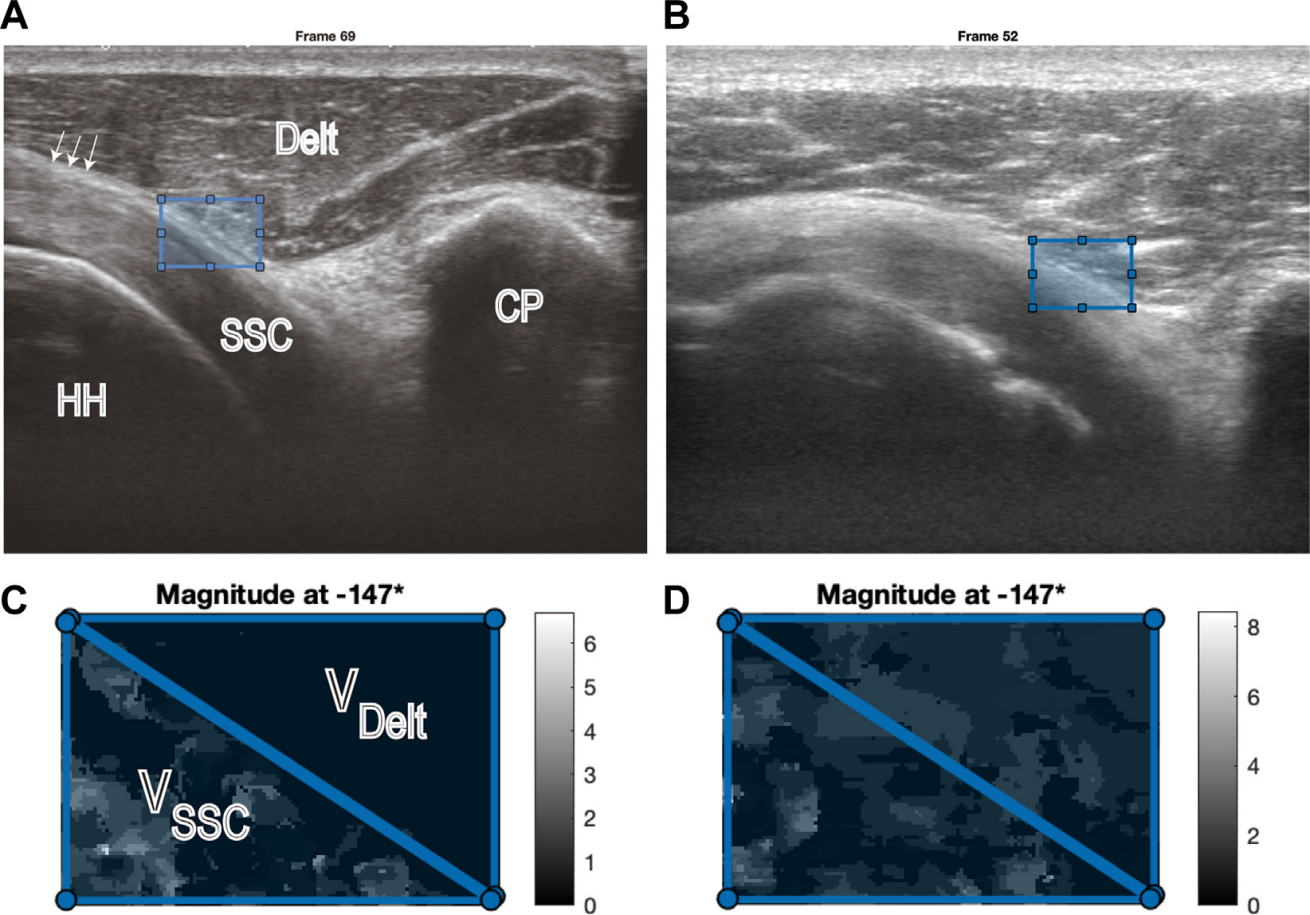
Examples of the speckle-tracking measurements for a low-DSAI (0.27) sample (**A**, **C**) and a high-DSAI (0.92) sample (**B**, **D**). (**A** and **B**) show image examples of the *Middle* frame of dynamic ultrasonography. The *blue rectangle* indicates the range of interest. *White arrows* indicate the coracohumeral ligament layer. (**C** and **D**) show velocity magnitude maps. Faster velocity areas are expressed in blighter colors. *Delt*, deltoid; *SSC*, subscapularis; *HH*, humeral head; *CP*, coracoid process; *V*_*Delt*_, average velocity of the deltoid area; *V*_*SSC*_, average velocity of the subscapularis area; *DSAI*, Deltoid–Subscapularis Adhesion Index (V_Delt_/V_SSC_).
4. The DSAI was repeatedly measured from the initial frame to the terminal frame by calculating the V_Delt_/V_SSC_.
5. The mean DSAI for the middle 30 frames of the motion was determined, and the average of the 2 measurements was used for statistical analysis.

Technically, the high DSAI value means that there is almost no difference in the velocity of the deltoid and the SSC observed with dynamic ultrasonography. Therefore, the value of V_Delt_/V_SSC_ is approaching unity. On the other hand, low DSAI is defined as occurring significant differences between the velocity of the deltoid and the SSC. In this case, the value of V_Delt_/V_SSC_ is approaching zero.

**Figure 3:**
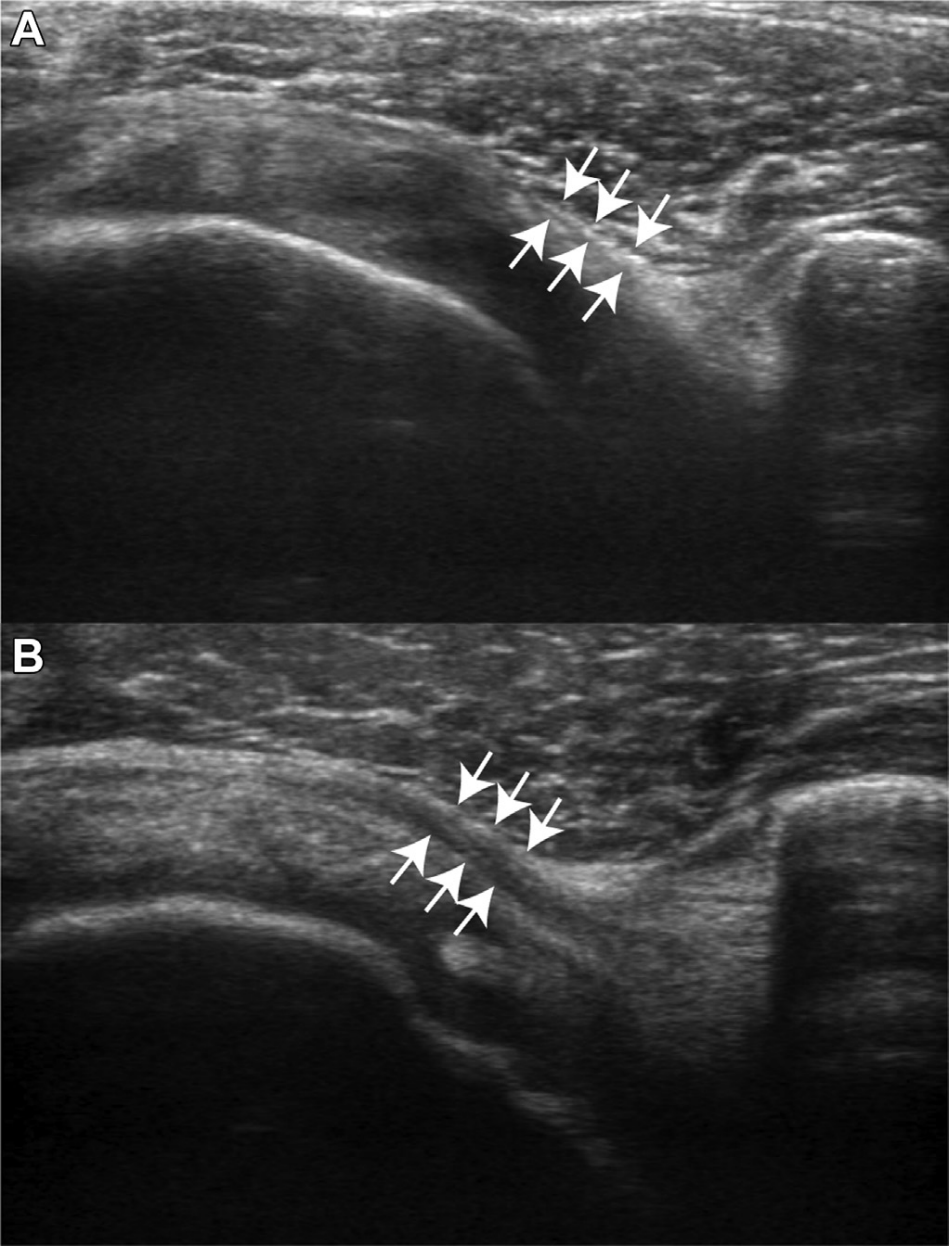
Samples of thin (**A**) and thick (**B**) coracohumeral ligament. The space between the *white arrows* presents the thickness of the coracohumeral ligament layer.

The CHL thickness was measured using the same images as those used for the DSAI calculation using the following procedures. Figure 3 presents typical thin and thick CHL images.

1. A video was played, and the CHL layer was confirmed.
2. The thickest point of the CHL was measured in the neutral rotation position corresponding to the terminal frame.

Shoulder external rotation and flexion ROM were measured using a universal goniometer in the supine position. External rotation ROM is defined as the angle between the forearm and perpendicular to the floor when the patient moves their arm outward with an arm at the side and 90° elbow flexion. Shoulder flexion ROM is defined as the angle between the arm and trunk when the patient moves their arm straight up with elbow extended.

### Statistical analysis

We used intraclass correlation coefficients (ICC) to confirm the reliability of DSAI measurements. We analyzed the DSAI of 70 shoulders in preoperative cases twice and calculated intrarater reliability. To calculate the inter-rater reliability, 2 independent examiners analyzed identical video sequences.

To compare basic information between pathological groups, we used the independent sample *t*-test for continuous values and the χ-square test for categorical data. Ultrasound assessments and ROMs were compared between the diagnostic groups and both sides of the shoulders (4-group comparison), and 1-way analysis of variance was used for comparison. The relationship between shoulder ROM, DSAI, and CHL thickness was confirmed by the diagnostic groups using Pearson’s correlation coefficient. In addition, multiple linear regression analysis was performed with external rotation ROM as the dependent variable and CHL thickness and DSAI as independent variables. We used R for Windows (R Development Core Team version 4.0.1; R Foundation for Statistical Computing, Vienna, Austria) for statistical calculations. The significance level was set at 5%.

We also performed power analysis to determine the sample size using G∗Power software (version 3.1; Heinrich Heine Universität, Düsseldorf, Germany) with an α error = 0.05, power = 0.8, and Cohen f = 0.8. Cohen f was estimated based on previous research, which compared the CHL thickness between FS, painful shoulder, and control.^13^^,^^22^ The minimum sample size for the 1-way analysis of variance was 6 for each group.

## Results

### Intrarater and inter-rater observer reliability of DSAI measurement

The ICC (1, 1), which represents the reliability of the intrarater observer was 0.85 (95% confidence interval: 0.77-0.90). The ICC (2,1) that represents the reliability of the inter-rater observer was 0.91 (95% confidence interval: 0.65-0.97).

### Participants enrollment

**Figure 4:**
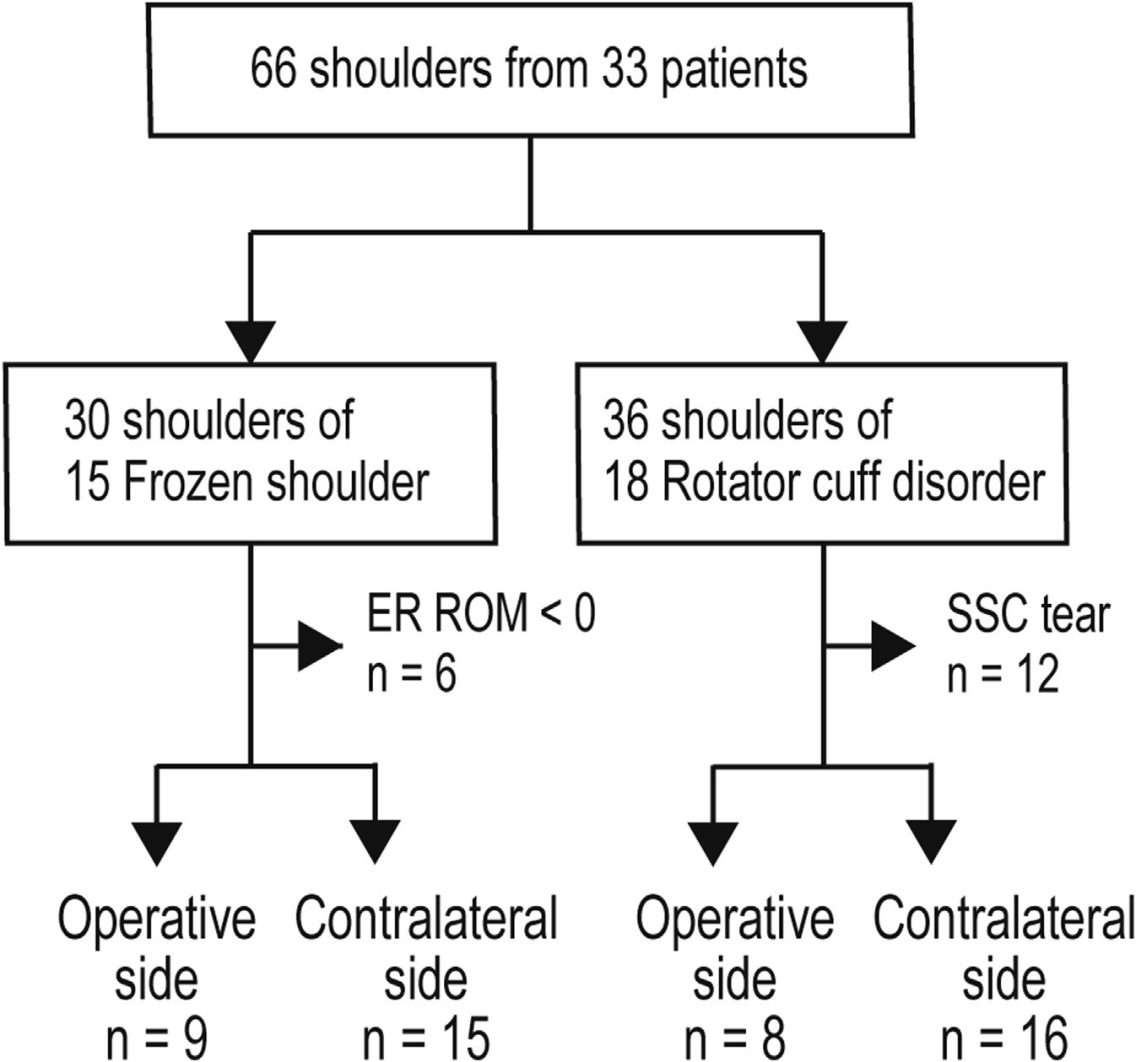
Flowchart of the enrollment process of study participants. Of the successive 47 patients who need operative care, 33 patients meet the diagnosis criteria of frozen shoulder or rotator cuff disorder. The subsequent allocation is illustrated.

Twenty-four shoulders of FS patients (9 operative and 15 contralateral side) and 24 shoulders of RCD patients (8 operative and 16 contralateral side) were enrolled for the statistical analysis. A flowchart of the enrollment process is shown in Figure 4.

### Intergroup comparison

The results of the group comparisons of basic information, ultrasound assessments, and ROMs are shown in Table I. Our CHL thickness measurements produced a similar thickness range as in a previous study with a cutoff value of 0.7 mm for diagnosing FS.^22^

**Table I:** Group comparisons for basic information, ultrasound outcome, and shoulder ROM.

Demographic data			
	FS (N = 15)	RCD (N = 18)	*P* value
Age (y)	53.3 ± 6.4	67.4 ± 10.6	*<.01*
Sex (Female)	6/15	9/18	.57
Height (cm)	165 ± 9.47	163 ± 9.96	.43
Weight (kg)	68.4 ± 16.4	62.1 ± 13.6	.25
Body mass index	24.8 ± 5.02	23.3 ± 3.5	.32
Symptom duration (mo)	5.33 ± 3.87	13.8 ± 14.4	*.03*

Ultrasound assessment and shoulder range of motion					
	OS (N = 9)	CS (N = 15)	OS (N = 8)	CS (n = 16)	
DSAI	0.58 ± 0.16	0.57 ± 0.19	0.91 ± 0.24†	0.64 ± 0.29	*<.01*
CHL thickness (mm)	1.00 ± 0.31∗	0.68 ± 0.29	0.81 ± 0.23	0.59 ± 0.21	*<.01*
External rotation (°)	17.2 ± 9.4∗	49.0 ± 14.3	30.6 ± 15.9∗	59.1 ± 19.3	*<.01*
Flexion (°)	105 ± 24.6†	157 ± 23.2	144 ± 15.1	163 ± 12.3	*<.01*

*FS*, frozen shoulder; *CHL*, coracohumeral ligament; *DSAI*, Deltoid–Subscapularis Adhesion Index (V_Delt_/V_SSC_); *RCD*, rotator cuff disorder; *ROM*, range of motion; *OS*, operation side; *CS*, contralateral side.

Data are presented as mean ± standard deviation. *P*-values below the significance level of .05 are italicized.

∗ Significant difference from both contralateral side groups.

† Significant difference from all other groups.

### Relationship between ultrasound assessments and shoulder ROM

**Figure 5:**
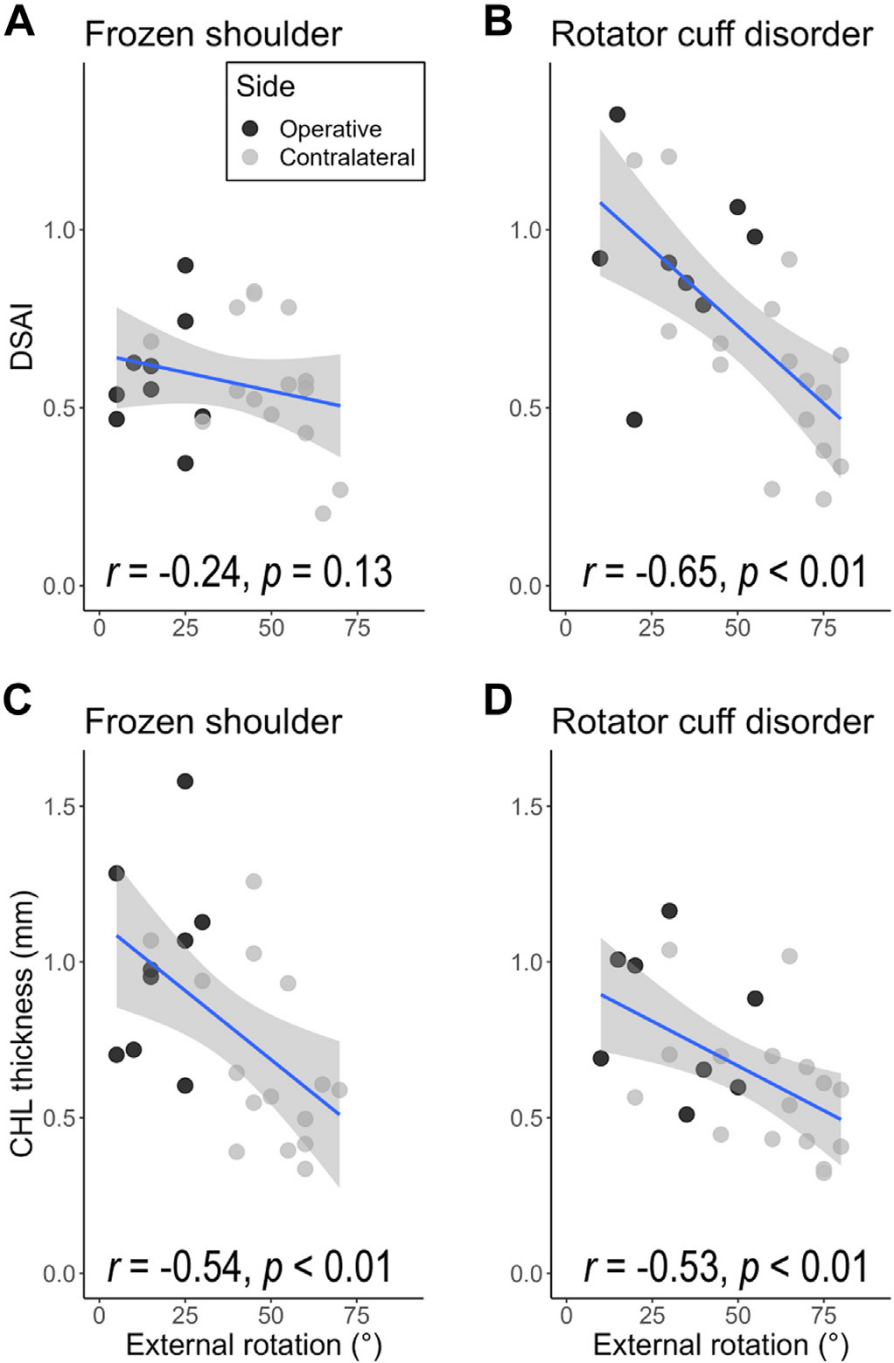
The results of frozen shoulder are shown in the left hand (**A**, **C**), and those of rotator cuff disorder are shown in the right hand (**B**, **D**). Upper plots (**A**, **B**) show DSAI against External rotation ROM. Lower plots (**C**, **D**) show CHL thickness against external rotation ROM. Pearson’s correlation coefficients are shown below the plots. Subsequent linear regression analysis revealed that CHL thickness is not a significant predictor of external rotation ROM in rotator cuff disorder group. *ROM*, range of motion; *CHL*, coracohumeral ligament; *DSAI*, Deltoid–Subscapularis Adhesion Index (V_Delt_/V_SSC_).

Figure 5 shows a scatter plot representing the DSAI and CHL thicknesses against the external rotation ROM. Multiple linear regression analysis reveals that only DSAI is a significant predictor for external rotation ROM in RCD (adjusted R^2^ = 0.44, F = 10.1, *P* < .01, t = −2.9), while CHL thickness is a significant predictor for external rotation ROM in FS (adjusted R^2^ = 0.28, F = 5.5, *P* = .01, t = −3.0).

## Discussion

The current evaluation method for adhesions between the shoulder muscles showed good reliability. Dynamic ultrasonography provides valuable information regarding tissue displacement, which cannot be obtained from static images. However, it should be noted that acquiring high-quality image sequences is challenging, and the motion task in this experiment has several limitations.

The participants were required to move their shoulders by themselves to avoid guarding and compensatory movements. In a pilot study, we used a device to navigate the movement passively, but it caused unintended movements such as scapular retraction, resulting in image artifacts. In addition, we must set a motion range that minimizes the scapular movement. These settings are still in their early stages and require further study.

Despite these limitations, this method is reliable, does not require specialized equipment, and can easily be adapted for daily use. With minor revisions, it can be used to evaluate other parts of the shoulder joint or even other joints. Although there is room for improvement, we confirmed the reliability of the current evaluation method and further research would make it more robust and practical.

Our secondary purpose was to determine the relationship between adhesion severity and shoulder ROM restrictions. We found that adhesions between the SSC and deltoid were negatively correlated with shoulder ROM. In addition, this association was observed only in the RCD group. Conversely, CHL thickness was an independent cause of ROM restriction in the FS group, whereas this was not a significant predictor of ROM in the RCD group.

These results suggest that the primary causes of ROM restriction are different between FS and RCD. CHL thickening can cause severe ROM restriction in FS, and a previous study showed that RCD is rarely observed in patients with severe and multidirectional ROM restrictions.^23^ On the other hand, RCD can be observed with severe adhesion. The adhesion between SSC and deltoid which results in ROM restrictions is probably induced by the inflammation of the rotator cuff tendon. Considering adhesions as the cause of ROM restriction may resolve confusion in the diagnosis and interpretation of clinical data on FS.

The consensus paper of American Shoulder and Elbow Surgeons defined FS in 2011 as "functional restriction of both active and passive shoulder motion for which radiographs of the glenohumeral joint are essentially unremarkable except for the possible presence of osteopenia or calcific tendonitis."^24^ They defined RCD-related ROM restrictions as secondary types of frozen shoulder, and our results support this classification. Although this classification has not been a gold standard, future studies should investigate FS according to subtypes that may have different etiological factors.

In many cases, FS is treated conservatively with physical therapy, intrajoint corticosteroid injection, and oral medication.^15^ A previous study found that mobilization methods affect the magnitude of wrist flexor tendon sliding relative to the surrounding tissues.^16^ Another study showed that soft tissue mobilization and proprioceptive neuromuscular facilitation (a stretching technique) immediately increased external rotation ROM^7^ These findings suggest that the rehabilitation of adhesions is effective. In addition, in operative cases of RCD with contracture, the subdeltoid region needs careful observation and release if adhesion occurs.

In contrast, refractory FS cases are often treated by surgery.^14^ However, few prognostic factors other than diabetes are available for refractory shoulder ROM restriction other than diabetes.^4^ Primary FS with a low DSAI and severe CHL thickening, which indicates less muscle involvement, can be an indication of refractory FS. Further studies are needed to better understand the classification of FS and optimize treatment selection.

Our study has certain limitations. In addition to the previously mentioned limitations of motion tasks, it is unclear whether the speed and range of shoulder movement could affect DSAI evaluation. Second, we excluded cases of full-thickness SSC tears and extremely severe contractures. Additional verification is required to determine whether the results of this study can be applied to these cases. Third, items such as duration of treatment could not be controlled in this study because details of the previous treatment history of patients referred to our hospital for surgery from other hospitals were not available. Finally, we could not control some information such as age, disease duration, and comorbidities because there were participants in whom both sides or only 1 side of the shoulders were included.

## Conclusion

The measurement of muscle stretched velocity by speckle tracking is valid, and the reliability of the current method is adequate. It also has good potential for clinical use. This study suggests that the dominant causes of ROM restrictions differ between primary and secondary (RCD-related) FS. Primary FS is often accompanied by CHL thickening, and adhesions between SSC and deltoid are more likely to occur in RCD-related FS. Further research is necessary to improve the evaluation method and to investigate the better classification of FS and intervention effectiveness for specific etiologic factors.

## Disclaimers:

Funding: There was no external funding for this study. The part of this study was supported by the 10.13039/100016987Research Center for Biomedical Engineering.

Conflicts of interest: The authors, their immediate families, and any research foundation with which they are affiliated have not received any financial payments or other benefits from any commercial entity related to the subject of this article.
